# Lung colony formation: a selective cloning process for lung-colony-forming ability.

**DOI:** 10.1038/bjc.1979.31

**Published:** 1979-02

**Authors:** N. Suzuki, H. R. Withers


*Br. J. Cancer* (1979) **39**, 196

***Short Communication***

### LUNG COLONY FORMATION: A SELECTIVE CLONING PROCESS FOR LUNG-COLONY-FORMING ABILITY

N. SUZUKI AND H. R. WITHERS

*From the Section of Experimental Radiotherapy, The University of Texas System Cancer Center, M. D. Anderson Hospital and Tumor Institute, Houston, Texas, U.S.A.*

Received 21 June 1978 Accepted 2 November 1978

METASTASIS is a phenomenon consisting of many steps, including release of cells from the tumour, circulation of released cells and their lodging at distant sites, and growth where these new sites are favourable. Both tumour-cell and host factors could be involved and interact in all these steps (Zeidman, 1957; Rubin & Green, 1968; Thompson, 1976). Metastatic foci are formed at low frequency in spite of the release of many malignant cells into the circulation (Engel, 1959; Roberts *et al.*, 1961) or despite i.v. injection of many living cells (Iwasaki, 1915; Takahashi, 1915; Warren & Davis, 1934.) Our working hypothesis to help explain this inefficiency of metastasis has been as follows: tumours vary with respect to their karyotype or malignant characteristic (Makino, 1957; Foulds, 1969; Nowell, 1976) so that cells within a tumour may be heterogeneous in various characteristics including malignant properties. Metastasis may require properties other than local growth ability, and variants with different potentials for metastatic growth may evolve during tumour development and progression. For metastatic growth, variants must meet the requirements for both dissemination and growth in the new tissue environment. Thus, metastasis could involve selective cloning. We have isolated clones from a mouse fibrosarcoma and characterized some of their cell characteristics, including their malignant potential. Plating efficiency (Suzuki & Withers, 1978*a*) DNA content (Suzuki *et al.*, 1977*b*) cell volume and lung-colony-forming efficiency (LCFE) (Suzuki & Withers, 1978*a*; Suzuki *et al.*, 1978*b*) were heterogeneous among clones. These results are consistent with our working hypothesis. In the experiments reported here, the LCFE of cells cultured from secondary lung nodules was compared with that of cells cultured from subcutaneous tumours or with that of the original cells (FSA 1233) in culture.

FSA 1233 is one of the clones isolated from a mouse fibrosarcoma (Suzuki & Withers, 1978*a*) and, after 5 subcultures, has been kept in liquid $N_2$. The cells were cultured in McCoy's 5A medium as modified by Hsu, containing 20% foetal calf serum. Cell suspensions from secondary lung nodules or subcutaneous tumours were made 25 days after injection, using trypsin and DNase I (Suzuki & Withers, 1978*a*). These cells were grown in culture for 10–20 days (3 subcultures) prior to i.v. injection into the tail veins of mice. No significant normal-cell contamination was observed by flow microfluorometry after this period of culture.

LCFE is affected by several factors: cell-cycle stage (Suzuki *et al.*, 1977*a*) the *in vitro* growth conditions prior to i.v. inoculation (Bosmann & Lione, 1974) and

Correspondence to: N. Suzuki, M.D., Ph.D., Section of Experimental Radiotherapy, The University of Texas System Cancer Center, M. D. Anderson Hospital and Tumor Institute, 6723 Bertner Avenue, Houston Texas 77030, U.S.A.

TABLE.—*Mean lung colonies/mouse ± s.e. (based on* 10–20 *mice) when inoculated with cells cultured from lung nodules or tumours*

| | Cells | % PE* | Cells injected/mouse† | | | |
|---|---|---|---|---|---|---|
| | | | $1\cdot1\times10^4$ | $3\cdot3\times10^4$ | $5\times10^4$ | $10^5$ |
| Exp. 1 | The original cells (FSA 1233) | 6·8±1·4 | 0·4±0·2 | 0·9±0·3 | | 12·0±3·0 |
| | A tumour (T1) | 9·1±0·7 | | | 3·7±1·0 | 9·7±2·4 |
| | A lung nodule (LC1) | 8·5±1·8 | | | | 157±15 |
| Exp. 2 | A tumour (T2) | 11·6±1·3 | | | 4·8±1·9 | 18·3±5·2 |
| | A lung nodule (LC1) | 9·3±1·0 | 11·2±2·3 | 62·7±12·2 | | 207±19 |
| Exp. 3 | The original cells (FSA 1233) | 51·9±6·2 | | | | 22·7±5·8 |
| | Mixed lung nodules (LM1) | 28·4±2·6 | | | | 182±18 |

* Mean±s.e. of 3–8 tubes. Plating efficiency was determined in screw-capped culture tubes by soft-agar cloning (Suzuki & Okada, 1976).

† Single-cell suspensions of $10^4$–$10^5$ cells in 0·5 ml of medium were injected into tail veins of unirradiated mice without addition of microspheres or heavily irradiated cells. Mice were killed 19 days after injection and lungs were removed and fixed in Bouin's solution. Colonies were counted macroscopically (Suzuki *et al.*, 1977*a*; Suzuki *et al.*, 1978*b*).

whether the cells were prepared from tumours or *in vitro* cultures (Suzuki & Withers, 1978*a*). In these experiments, cells in the late log-phase of growth in the third subculture were used for both LCFE and host survival-time experiments. Late-log-phase cultures were trypsinized, washed by centrifugation, and resuspended in medium. Cells were counted with a Coulter counter (model ZBI) and a multichannel analyser (Channelyzer II) (Suzuki *et al.*, 1977*a*; Suzuki & Withers, 1978*a*). The suspensions were routinely checked for clumps and viability using a phase-contrast microscope. One of the features of the fibrosarcoma used is that it is easy to make single-cell suspensions without clumps (Suzuki & Withers, 1978*a*). C3Hf/Bu male mice, 8–10 weeks old, were obtained from our specific-pathogen-free breeding colony.

The Table describes details of the experiments and the results, and the Figure summarizes these results. Experiment 1 (Table) shows that cells from a single nodule in a lung (LC1) had a higher LCFE than the original FSA 1233 cells, whereas cells from a subcutaneous tumour (T1) had an LCFE similar to that of the original FSA 1233 cells. Experiment 2 was a repetition of Experiment 1. Experiment 3 shows that cells from a mixed culture of all lung colonies from one mouse (LM1) had a higher LCFE than the original FSA 1233 cells. The *in vitro* plating efficiencies of these cells are also shown in the Table, but differences in PE's do not explain the difference of LCFE among cells from nodules, tumours or the original FSA 1233 culture.

These results were interpreted as follows. The higher LCFE of cells from lung nodules was not related solely to growth *in vivo*, since growth as subcutaneous tumours did not yield cells of higher LCFE. The fact that cells from a single nodule or from a mixture of metastatic nodules showed similar high levels of LCFE suggests that metastasis could be a process in which variants from the initial primary tumour showing increased metastatic growth ability are selectively cloned for a particular metastatic site.

When 10 mice per group were inoculated s.c. with $10^6$ cells per locus at 4 loci, the days for 50% survival of the hosts were 33 and 38 days for FSA 1233, and 33 and 37 days for a mixture of cells from several lung nodules (LM1). The results of 2 separate experiments showed no difference between the survival time of mice inoculated s.c. with either LM1 or the

FIGURE.—Lung-colony-forming ability of cells cultured *in vitro* from a single metastatic lung nodule (△), a mixture of lung nodules (▲), tumours growing s.c. (●), or the original FSA 1233 (○). Error bars show s.e. among 10–20 mice. (Same data as in Table.)

parental FSA 1233, despite the 10-fold greater artificial lung-colony-forming ability of the LMl cells. This indicates that some factors responsible for lung-colony formation are different from those involved in local subcutaneous growth.

The present experimental results agree with various clinical and experimental facts: the inefficiency of metastasis formation despite the presence of many malignant cells in the blood stream (Engel, 1959; Roberts *et al.*, 1961) tumour-specific distribution of metastatic foci, the "seed soil theory" of Paget (1889), and the development concept of malignancy which suggests heterogeneity of karyotype or malignant characteristics among cells in a tumour (Makino, 1957; Foulds, 1969; Nowell, 1976). Fidler (1973) has already isolated highly metastatic variants of B16 melanoma cells by 5 to 10 passages of the cells as lung metastases. B16 melanoma is a long-established line, whereas the clone used in these experiments was freshly isolated from a mouse fibrosarcoma. Our experiment showed that the selection of increased metastatic efficiency was specific to the organ in which the metastases were passaged, because s.c. passage did not enhance lung-colony-forming efficiency.

In summary, lung-colony formation was a selective cloning process for factors involved in lung-colony formation.

We thank Ms Marcia W. Koehler for excellent technical assistance with the experiments, and for the preparation of this manuscript. We are also grateful to Larry Wilborn and his staff for the supply and care of the animals used in these experiments.

This investigation was supported in part by research grants CA-06294 and CA-11138, awarded by the National Cancer Institute, DHEW.

Animals used in this study were maintained in facilities approved by the American Association for Accreditation of Laboratory Animal Care, and in accordance with current United States Department of Agriculture and Department of Health, Education, and Welfare, National Institutes of Health regulations and standards.

### REFERENCES

BOSMANN, H. B. & LIONE, A. (1974) Capacity for tumour cell implantation as a function of *in vitro* cell density. *Biochem. Biophys. Res. Commun.*, **61**, 564.

ENGEL, H. C. (1959) Cancer cells in the blood. *Ann. Surg.*, **149**, 457.

FIDLER, I. J. (1973) Selection of successive tumour lines for metastasis. *Nature* (*New Biol.*), **242**, 148.

FOULDS, L. (1969) *Neoplastic Developments. I*, London: Academic Press. p. 41.

IWASAKI, T. (1915) Histological and experimental observations on the destruction of tumor cells in the blood vessels. *J. Pathol.*, **20**, 85.

MAKINO, S. (1957) The chromosome cytology of the ascites tumors of rats with special reference to the concept of the stem cell. *Int. Rev. Cyt.*, **6**, 26.

NOWELL, P. C. (1976) The clonal evolution of tumor cell populations. *Science*, **194**, 23.

PAGET, S. (1889) The distribution of secondary growths in cancer of the breast. *Lancet*, i, 571.

ROBERTS, S., JOHNASSON, O., LONG, L., MCGRATH, R., MCDREW, E. A. & COLE, W. H. (1961) Clinical significance of cancer cells in the circulating blood: two to five year survival. *Ann. Surg.*, **154**, 362.

RUBIN, P. & GREEN, J. (1968) *Solitary Metastases.* Springfield: Charles C. Thomas.

SUZUKI, N. & OKADA, S. (1976) Isolation of nutrient deficient mutants and quantitative mutation assay by reversion of alanine requiring L5178Y cells. *Mutation Res.*, **34**, 489.

SUZUKI, N., FRAPART, M., GRDINA, D. J., MEISTRICH, M. L. & WITHERS, H. R. (1977*a*) Cell cycle dependency of metastatic lung colony formation. *Cancer Res.*, **37**, 3690.

SUZUKI, N., WITHERS, H. R. & LEE, L. Y. (1977*b*) Variability of DNA content of murine fibrosarcoma cells, *Nature*, **269**, 531.

SUZUKI, N. & WITHERS, H. R. (1978*a*) Isolation from a murine fibrosarcoma of cell lines with enhanced plating efficiency *in vitro*. *J. Natl Cancer Inst.*, **60**, 179.

SUZUKI, N., WITHERS, H. R. & KOEHLER, M. W. (1978*b*) Heterogeneity and variability of artificial lung colony-forming ability among clones from mouse fibrosarcoma. *Cancer Res.*, **38**, 3349.

TAKAHASHI, M. (1915) An experimental study of metastasis. *J. Pathol.*, **20**, 1.

THOMPSON, S. C. (1976) Effect of age and sex on lung-colony-forming efficiency of injected mouse tumour cells. *Br. J. Cancer*, **34**, 566.

WARREN, S. & DAVIS, A. H. (1934) Studies on tumor metastasis. V. The metastases of carcinoma to the spleen. *Am. J. Cancer*, **21**, 517.

ZEIDMAN, I. (1957) Metastasis: a review of recent advances. *Cancer Res.*, **17**, 157.


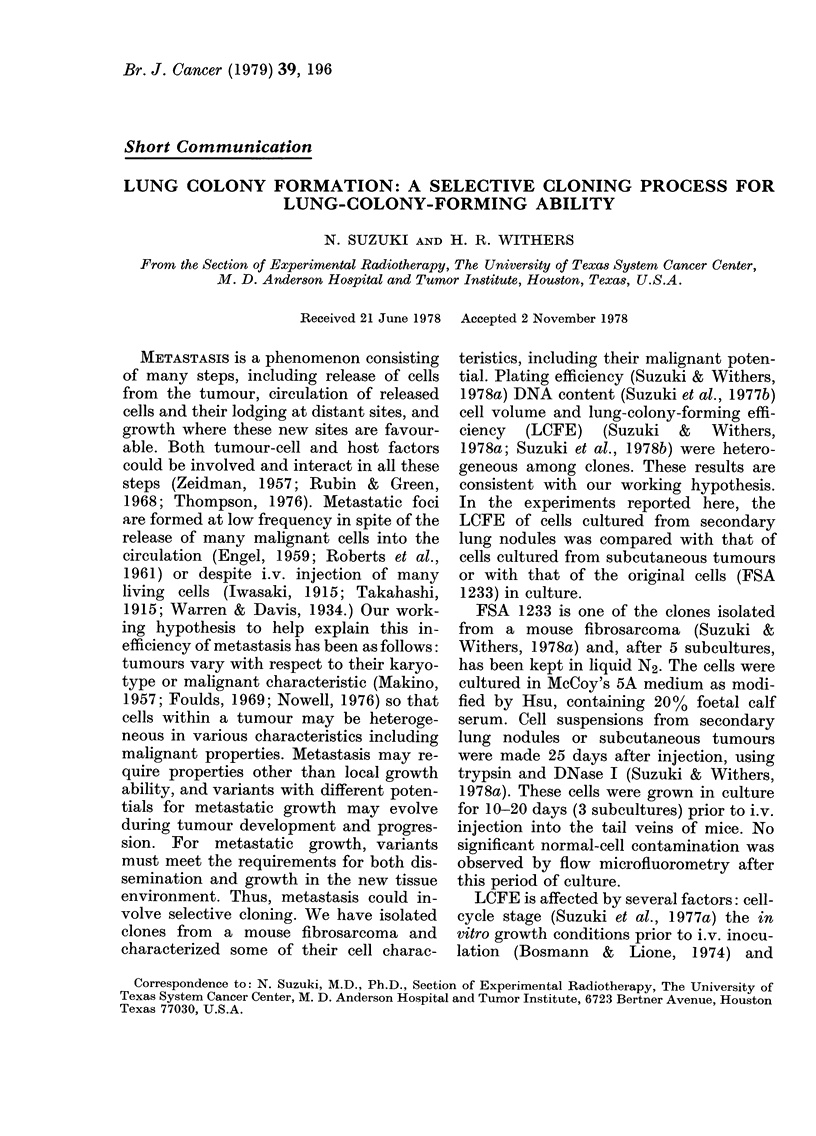

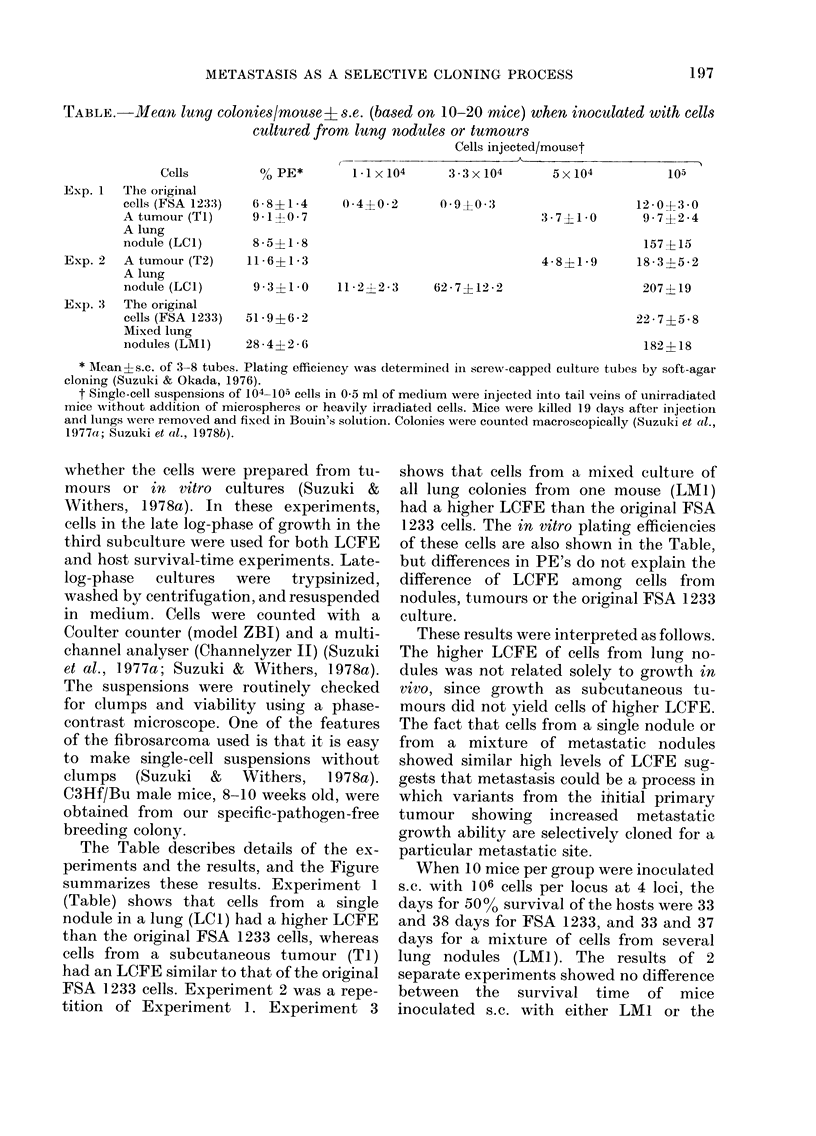

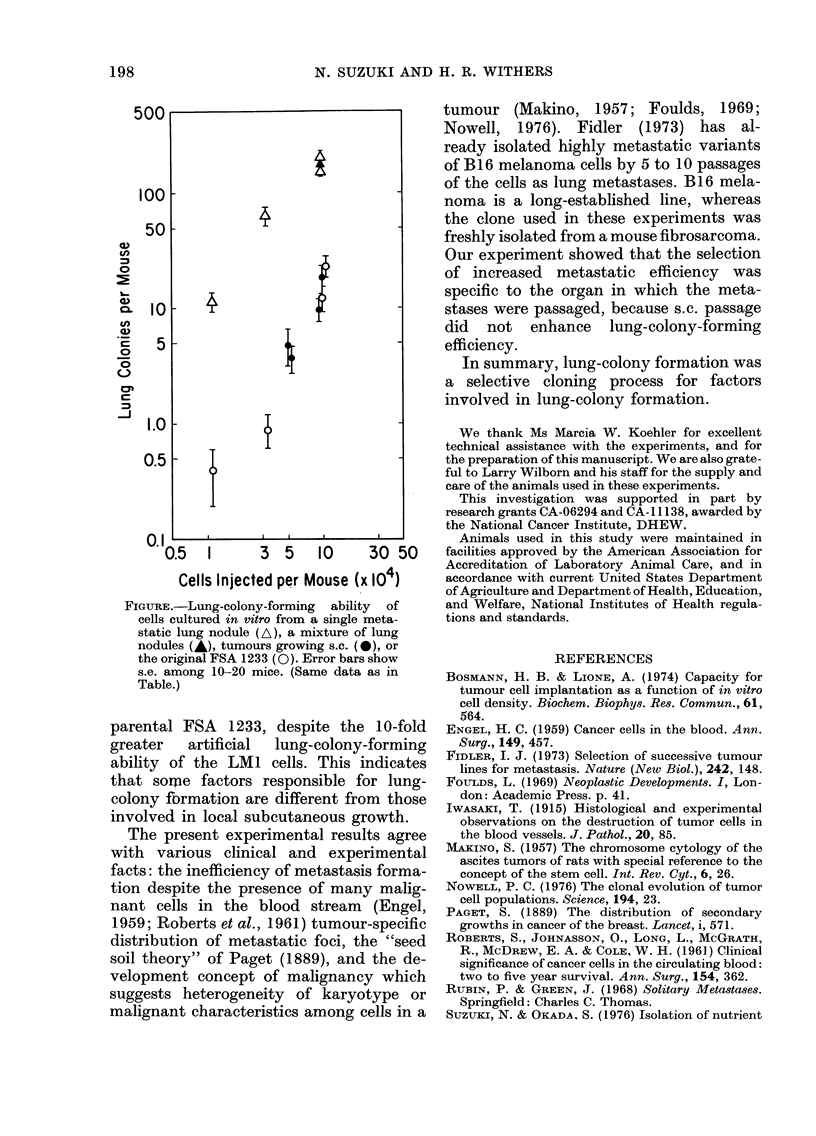

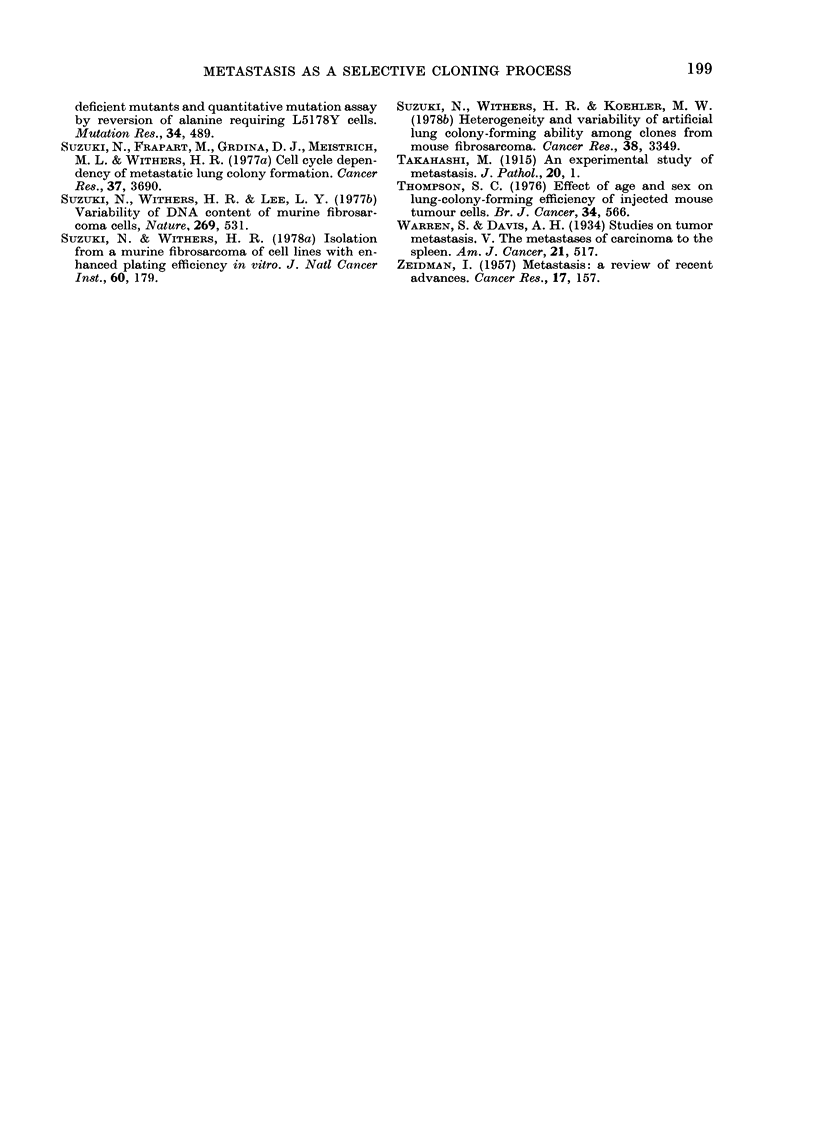


## References

[OCR_00310] Bosmann H. B., Lione A. (1974). Capacity for tumor cell implantation as a function of in vitro cell density.. Biochem Biophys Res Commun.

[OCR_00316] ENGELL H. C. (1959). Cancer cells in the blood; a five to nine year follow up study.. Ann Surg.

[OCR_00320] Fidler I. J. (1973). Selection of successive tumour lines for metastasis.. Nat New Biol.

[OCR_00337] Nowell P. C. (1976). The clonal evolution of tumor cell populations.. Science.

[OCR_00345] ROBERTS S., JONASSON O LONG L., McGRATH R., McGREW E. A., COLE W. H. (1961). Clinical significance of cancer cells in the circulating blood: two- to five-year survival.. Ann Surg.

[OCR_00365] Suzuki N., Frapart M., Grdina D. J., Meistrich M. L., Withers H. R. (1977). Cell cycle dependency of metastatic lung colony formation.. Cancer Res.

[OCR_00355] Suzuki N., Okada S. (1976). Isolation of nutrient deficient mutants and quantitative mutation assay by reversion of alanine-requiring L5178Y cells.. Mutat Res.

[OCR_00374] Suzuki N., Withers H. R. (1978). Isolation from a murine fibrosarcoma of cell lines with enhanced plating efficiency in vitro.. J Natl Cancer Inst.

[OCR_00380] Suzuki N., Withers H. R., Koehler M. W. (1978). Heterogeneity and variability of artificial lung colony-forming ability among clones from mouse fibrosarcoma.. Cancer Res.

[OCR_00369] Suzuki N., Withers H. R., Lee L. Y. (1977). Variability of DNA content of murine fibrosarcoma cells.. Nature.

[OCR_00390] Thompson S. C. (1976). Effect of age and sex on lung-colony-forming efficiency of injected mouse tumour cells.. Br J Cancer.

[OCR_00400] ZEIDMAN I. (1957). Metastasis: a review of recent advances.. Cancer Res.

